# A Bibliometric Analysis of 3D Printing in Personalized Medicine Research from 2012 to 2022

**DOI:** 10.3390/ph16111521

**Published:** 2023-10-26

**Authors:** Aile Xue, Wenjie Li, Wenxiu Tian, Minyue Zheng, Lan Shen, Yanlong Hong

**Affiliations:** 1 Shanghai Innovation Center of TCM Health Service, Shanghai University of Traditional Chinese Medicine, No. 1200, Cai-Lun Road, Pudong District, Shanghai 201203, China; 22022550@shutcm.edu.cn (A.X.); liwenjie@shutcm.edu.cn (W.L.); 22021554@shutcm.edu.cn (W.T.); zmy2290677028@126.com (M.Z.); 2 College of Chinese Materia Medica, Shanghai University of Traditional Chinese Medicine, No. 1200, Cai-Lun Road, Pudong District, Shanghai 201203, China

**Keywords:** 3D printing, personalized, pharmaceutical preparations, drug release, drug dosage forms, bibliometric analysis

## Abstract

In recent years, the 3D printing of personalized drug formulations has attracted the attention of medical practitioners and academics. However, there is a lack of data-based analyses on the hotspots and trends of research in this field. Therefore, in this study, we performed a bibliometric analysis to summarize the 3D printing research in the field of personalized drug formulation from 2012 to 2022. This study was based on the Web of Science Core Collection Database, and a total of 442 eligible publications were screened. Using VOSviewer and online websites for bibliometric analysis and scientific mapping, it was observed that annual publications have shown a significant growth trend over the last decade. The United Kingdom and the United States, which account for 45.5% of the total number of publications, are the main drivers of this field. The *International Journal of Pharmaceutics* and University College London are the most prolific and cited journals and institutions. The researchers with the most contributions are Basit, Abdul W. and Goyanes Alvaro. The keyword analysis concluded that the current research hotspots are “drug release” and “drug dosage forms”. In conclusion, 3D printing has broad application prospects in the field of personalized drugs, which will bring the pharmaceutical industry into a new era of innovation.

## 1. Introduction

In 3D printing (3DP), also known as “additive manufacturing”, computer-aided design (CAD) is used to construct objects via layer-by-layer printing [1,2,3], which is an emerging technology in the field of manufacturing, described as “manufacturing technology with the significance of industrial revolution”. In the late 1980s, various 3D printing technologies mushroomed [4]. In 1996, the world’s first pharmaceutical 3D printing company was established to introduce 3D printing technology into the traditional pharmaceutical field [5]. Recently, with the progress of industrial technology, 3D printing technology has been rapidly developed and is gradually being introduced in various fields, including construction, transportation, electronics, and medicine. In 2015, the first anti-epileptic drug Spritam was approved by the Food and Drug Administration (FDA), marking the official entry of 3D printing as a new technology in drug development and production [6]. In the past few years, 3D printing has been used to prepare various dosage forms of drugs, such as tablets, suppositories, microneedles, orally dispersible films, and capsules. The main technologies used include fused deposition modeling (FDM) [7,8,9,10,11], semi-solid extrusion technology (SSE) [12,13,14], direct powder extrusion (DPE) [15,16], inkjet printing (IP) [17,18,19], stereoscopic lithography (SLA) [6,20,21], and selective laser sintering (SLS) [22,23,24].

The traditional practice in medicine is to prescribe the same prescription to different patients with the same disease. This is appropriate for most people, but for some patients, it may be ineffective or there maybe be toxic side effects of the drug [25], and this method does not accommodate the need for clinical diversity. “Personalized medicine” was first introduced in 1999 [26,27]. The prescription of more precise drugs according to the different conditions, genetic traits, and physical conditions of patients has become a common quest for patients and doctors [2]. In addition, traditional pharmaceutical processes lack flexibility. As an emerging technology for personalized medicine, 3D printing offers a high degree of flexibility. By changing the geometry, size, and formulation of drugs in terms of their 3D design, different drug dosage forms can be printed to control drug release, meet the needs of personalized medicine therapy, and improve drug safety. Moreover, the 3D printing production process is more concise, allowing for on-demand production and improving pharmaceutical efficiency [28]. 

As a combination of “data science” and modern science, bibliometrics takes research trends, literature sources, journal contributions, authors, co-citations, and keywords as research objects, revealing the current status of a subject area more accurately and scientifically. First, in our study, bibliometric analysis is used to analyze the research trends of 3D printing in the field of personalized medicine in the past 10 years from a macro perspective and determine the annual growth of this research field and the most influential countries, institutions, journals, authors, and research hotspots. Based on the objective identification of research hotspots, we further outline the advantages and disadvantages of different technologies and the application of 3D printing in personalized drug dosage forms. Finally, we present the prospects and challenges of 3D printing in the field of personalized drug formulations.

## 2. Result

### 2.1. Trend and Annual Counts

**Figure 1:**
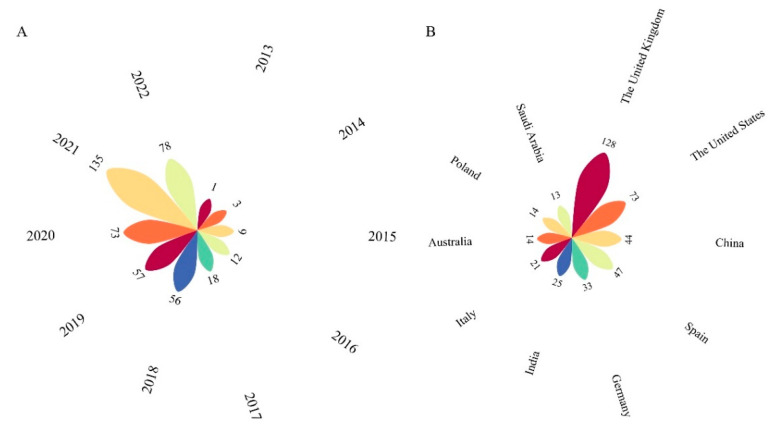
(**A**). The annual distribution of publications. (**B**). The top 10 countries in terms of publication quantities.

From 2012 to 2022, there were 442 research papers on 3D printing in personalized medicine, and the number of annual publications is shown in Figure 1. Based on the number of retrieved papers, 3D printing research in the field of personalization is in the early-development stage. According to the Web of Science Core Collection (WOSCC) database, these 442 articles have been cited 21,253 times, with an average citation frequency of 48.08 times. In 2015, the first antiepileptic drug Spritam was approved by the FDA [29], marking the formal entry of 3D printing into the field of drug development and production as a new technology with regulatory approval. This aroused the attention of researchers interested in 3D-printed drugs. As a result, the number of publications in 2016 and 2017 was more than two times that of the previous year. After several years of accumulated research, a vast number of researchers joined this field, and the number of annual publications reached more than 50 in 2018–2020. In January 2021, Triastek’s 3D-printed drug T19 received clinical trial approval from China’s National Medicinal Products Administration (NMPA) and IND approval from the FDA; it was the first 3D-printed drug to enter the registration and application stage in China. This sparked researchers’ interest in developing new drugs through 3D printing technology. In recent years, the demand for personalized medicine among patients has increased, and the development of new drugs has boosted researchers’ confidence in the use of 3D printing in the field of personalized medicine. The reason for the decline in the number of publications in 2022 may be related to the timing of the count, and there may still be some unpublished articles not included in the count for 2022. The simultaneous annual increase in the number of publications also suggests that the 3D printing of personalized medicines is a trend.

### 2.2. Contributions of Countries

**Figure 2:**
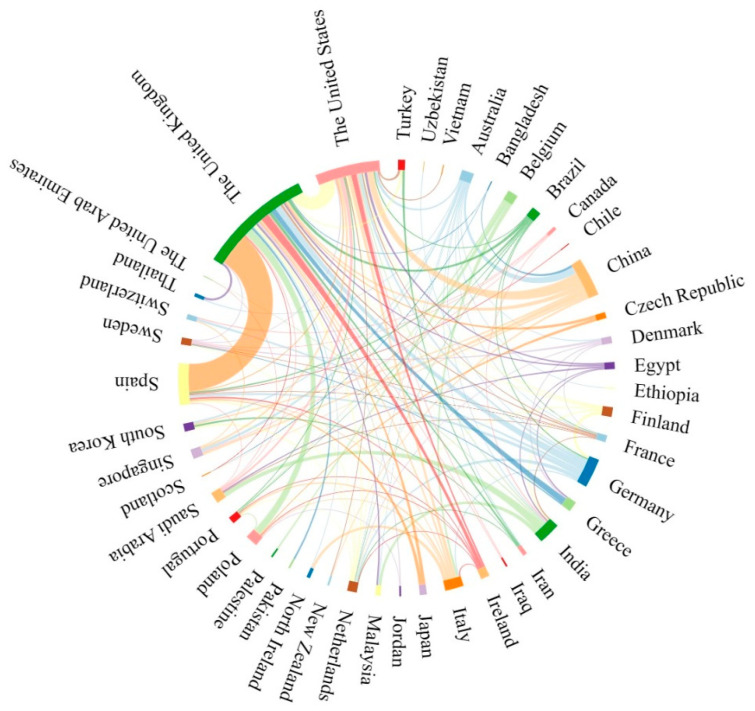
Publication volume and cooperation in different countries.

A total of 56 countries contributed to research in this field. The United Kingdom (UK) topped the list in terms of publication volume, followed by the United States (US), Spain, China, and Germany. These five countries accounted for 73.53% of the total number of publications. In Figure 2, the different colored lines represent different countries, and the lengths of the lines represent the number of publications; the longer the line is, the more publications there are. A connecting line between two line segments represents cooperation between countries, and the thickness of this connecting line represents the intensity of cooperation between countries. It can be seen that the United Kingdom has the strongest degree of cooperation with Spain and engages in a great deal of cooperation with the United States. Moreover, countries with more publications have more cooperative communication. In conclusion, cooperation is the key to development. Cooperation, interaction, and complementarity between different countries or institutions have far-reaching significance and can promote the development of this field.

### 2.3. Contributions of Journals

In Table 1, the top 10 journals that have contributed the most to 3D-printed personalized drug formulations are listed. At the top of the list are the *International Journal of Pharmaceutics* (109) and *Pharmaceutics* (74), which accounted for 41.40% of the total number of articles, far more than any other journal. In addition, the impact factors, i.e., quantitative indices used to evaluate the importance of the absolute or total citation frequency of these two journals, are 5.8 and 5.4, indicating that these two journals have a strong disciplinary influence and almost cover the latest advances in the field. Out of 358 journals, the *International Journal of Pharmacy* is the second-most-cited journal in the category “Pharmacy and Pharmacology”. It is the true home of pharmaceutical scientists who study the physical, chemical, and biological properties of devices and delivery systems for drugs, vaccines, and biologics, including their design, manufacture, and evaluation. In addition, the top ten journals have impact factors above 3.0, and most of the Journal Citation Reports (JCR) are in Q1 and Q2. The journal with the highest impact factor is *Advanced Drug Delivery Reviews*, which aims to provide a forum for the critical analysis of advanced drug and gene delivery systems and their applications in human and veterinary medicine.

### 2.4. Contributions of Institutions

**Figure 3:**
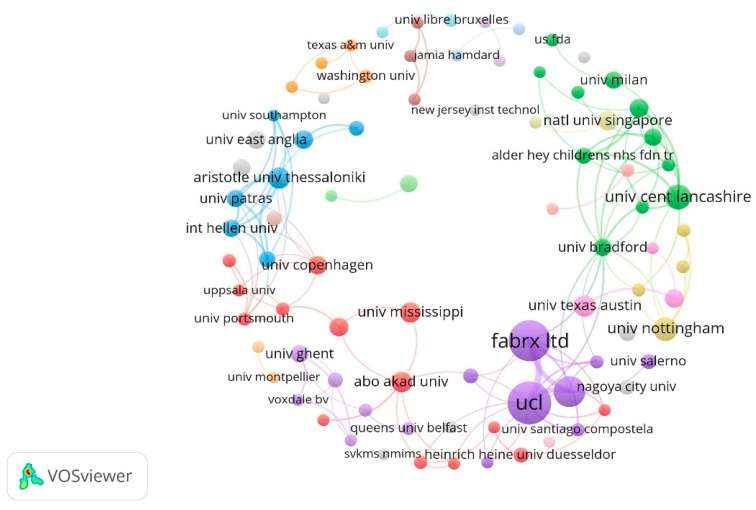
Co-authorship analysis of institutions based on the VOSviewer visualization map.

A total of 526 organizations have contributed to this field. Table 2 lists the 10 institutions with the most publications. In our VOSviewer analysis (Figure 3), we selected institutions that published more than three papers for analysis. Larger circles indicate that more papers have been published, and the lines between them indicate collaboration between institutions, with thicker lines indicating more collaboration. University College London (52), FabRx Ltd. (47), and Univ Santiago de Compostela (25) are the top three institutions in terms of the number of papers published, and they also collaborate more frequently with each other. FabRX Ltd. is a 3D printing company that developed the world’s first 3D printer for personalized medical printing, M3DIMAKER™. In addition, two of the founders of FabRx Ltd. are affiliated with University College London, and one is a professor at the University of Santiago de Compostela, so they have published more articles than anyone else; these include, for example, articles on the collaboration between the three organizations to print a multilayered polypill containing six medications using SLA technology [30], which, for the first time, proved the feasibility of SLA printing as an innovative platform for the production of multidrug therapies, facilitating the arrival of a new era of personalized multipills.

### 2.5. Contributions of Authors 

**Figure 4:**
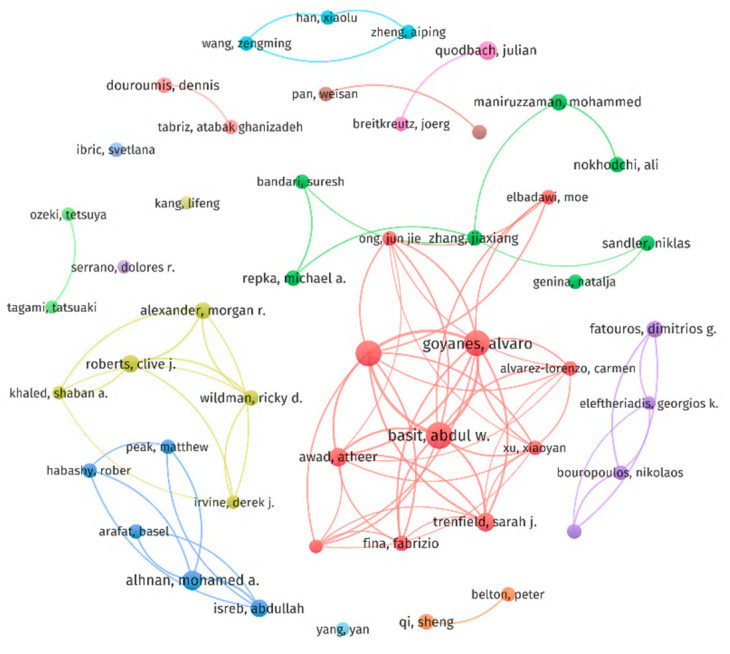
Co-authorship analysis regarding the analyzed authors based on the VOSviewer visualization map.

A total of 1663 scholars have contributed to research in this field. Basit, Abdul W., Goyanes Alvaro, and Gaisford Simon are in the top three, with 48, 46, and 43 papers published, respectively, all of whom are well known and influential scholars who collaborate greatly with each other. We chose authors with more than five publications for a VOSviewer analysis (Figure 4), where the circles represent different authors and the sizes of the circles represent the number of publications. The larger the circle, the greater the number of articles published. The lines between the circles represent collaborations. We can also see that most of the authors have their own collaborative networks with each other, dominated by the red clusters, where Basit, Abdul W., Goyanes Alvaro, and Gaisford Simon are the leaders. However, there are some authors who do not collaborate with other authors, which suggests that they are just entering the field and have yet to establish more collaborations with other researchers. As this technology improves, more researchers will begin working on 3D-printing-based personalized medicines to promote the development of more personalized medicine and precision medicine.

### 2.6. Keyword Analysis

**Figure 5:**
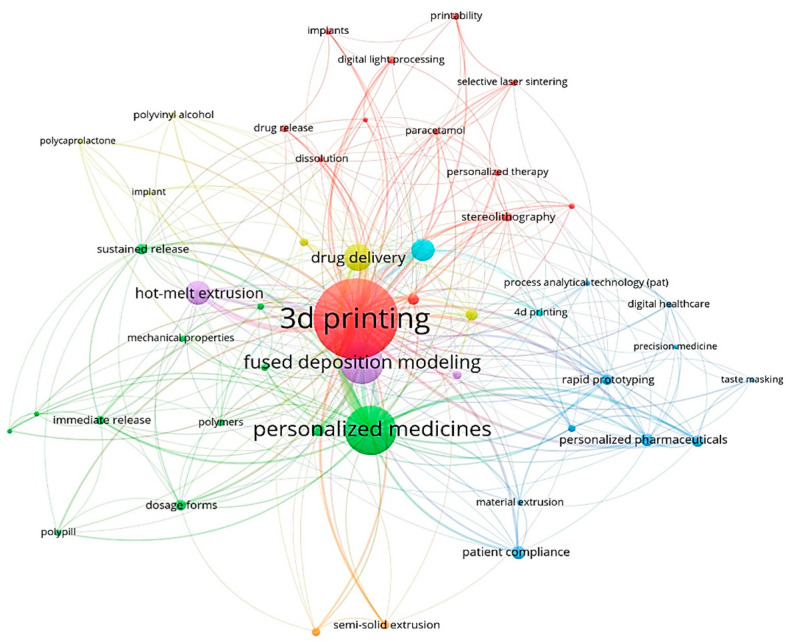
Analysis of keywords based on the VOSviewer visualization map.

After merging some keywords with the same meaning but different spellings, we used VOSviewer to analyze the author keywords that appeared more than five times (n = 49), and the results are shown in Figure 5. The colors in the figure represent the clusters. Again, five clusters were formed, with keywords serving as network nodes. Node size indicates the number of occurrences. The top five most used keywords were “personalized medicines”, “fused deposition modeling”, “drug delivery”, “hot-melt extrusion”, and “controlled release”. The connecting lines between the nodes indicate that the different keywords are closely related to each other. In addition to the first five keywords, “sustained release”, “drug release”, and “dosage forms” appear more frequently. It can be inferred that in recent years, the research hotspots of 3D-printed personalized drug formulations have mainly focused on the application of 3D printing technology, the development of dosage forms, and drug release, and FDM technology is a commonly used 3D printing technology in the field of personalized drug formulations.

### 2.7. Co-Citation of Cited References

**Figure 6:**
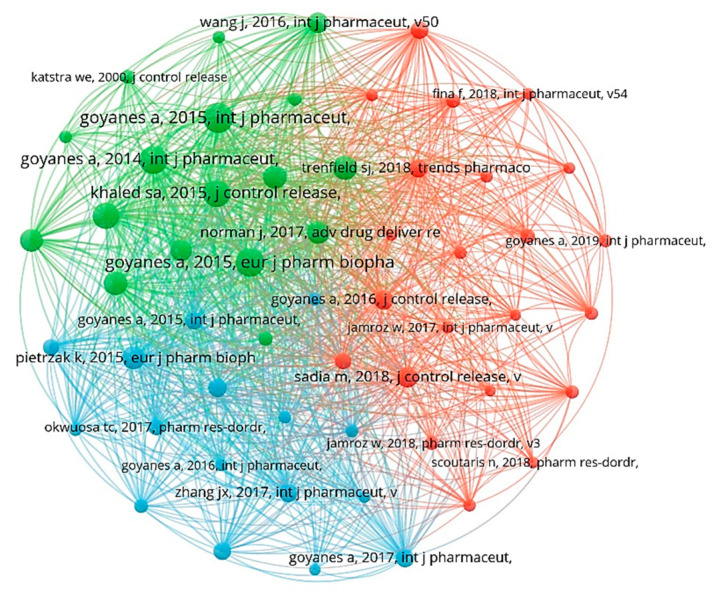
Analysis of co-citation of references based on the VOSviewer visualization map.

In this study, 442 documents were analyzed for the co-citation network, and a total of 55 documents with more than 50 citations were selected as co-citation network nodes, forming three clusters. As shown in Figure 6, the colors in the figure represent the clusters, and the node sizes represent citation frequency. By reading 55 articles, it was discovered that most of the literature in the green clusters was published in 2015, with most of the authors being more inclined to the theoretical research of 3D-printing-based personalized drugs, and these studies subsequently provided a theoretical basis and reference for researchers. Moreover, some innovative ideas can promote the depth of research in this field, and some of the literature has been cited more often. For example, Alvaro Goyanes and his team demonstrated that the release of tablets is independent of the surface area of a drug and depends on the surface-area-to-volume ratio (SA/V), which was determined using printing tablets with different geometries and by observing the rates of drug release [31]. This provided a reference for later researchers in developing different drug release rates via changing the geometries of drugs [32,33]. The red and blue clusters are mainly based on theoretical research on innovative topics such as technical method improvement and innovation [8,10,34]. We have listed the top 10 most-cited references (Table 3) that are most helpful to research in this field.

## 3. Discussion

### 3.1. Basic Information

We used bibliometric and visualization methods to analyze the use of 3D printing technologies in the field of personalized pharmaceutical formulations. By analyzing the relevant papers, we found that research in this area is on the rise, suggesting that 3D printing for personalized medicine is a major trend. The number of papers and citations produced by each institution and country are important indicators of research contributions in this area. The uneven development of research in different countries or regions is often mainly precipitated by a discipline’s level of development. In this research field, the UK, the US, and China have made outstanding contributions. The UK leads as the country with the most publications. Of all the research institutions, University College London, FabRx Ltd., and the Santiago de Compostela institution are the most active. The most prolific authors in the field are Basit, Abdul W.; Goyanes, Alvaro; and Gaisford, Simon. In addition, our study found that the countries, institutions, and authors that have contributed the most to the field share a common characteristic: cooperation and exchange; only through inter-institutional cooperation, transnational cooperation, and joint efforts can we contribute more to the research in this field and advance it, and such actions have become an international trend in conducting research.

### 3.2. Research Hotspots 

It has become the common pursuit of patients and doctors to provide more accurate medical treatments according to patients’ different conditions, genetic characteristics, and physical conditions. In our keyword reproduction analysis, we observed that FDM technology appears very frequently in this field, indicating that this technology is relatively common. Second, we observed a hot trend in the use of 3D printing in solid pharmaceutical dosage printing, mainly including the development of pharmaceutical dosage forms and the study of drug release. As an emerging personalized medicine technology, 3D printing has a high degree of flexibility. In addition to printing drugs that cannot be produced using traditional processes, researchers can print dosage forms suitable for different patients by changing a drug’s geometry, size, color, etc., through computer settings. At present, the development of pharmaceutical dosage forms mainly includes tablets, capsules, suppositories, implants, microneedles, etc., for which tablets are the most common dosage forms. Second, 3D printing can also be used to accurately control drug dosages and drug release rates via changing a drug’s formula, body/surface area ratio, filling density, etc., to achieve immediate release, sustained release, and controlled release and meet the needs of personalized treatment.

## 4. Materials and Methods

### 4.1. Data Collection 

Data from this study were extracted from the Web of Science Core Collection (WoSCC), (https://www.webofscience.com (accessed on 18 April 2023)). The search formula was set as follows: {[TS = (“3D printing”) AND [TS = (“personal*” OR “individ*”)] AND [TS = (“drug” OR “pharmaceutical” OR “medicine”)] AND PY = (2012–2022)}. Conditions were set for all articles published between 1 January 2012 and 31 December 2022. We read the titles and abstracts of the articles to eliminate anything irrelevant to the topic. Differences in the screening process were resolved through consultations between the submitters. The exclusion criteria were “3D-printed food”, “3D Bio-Printing”, and articles that mentioned 3D printing but did not focus on drugs. Results: A total of 442 pieces of data were included for follow-up analysis.

### 4.2. Data Analysis

In this study, we used VOSviewer1.6.16, Charticulator (https://charticulator.com/app/index.html (accessed on 24 April 2023)), and Chiplot (https://www.chiplot.online (accessed on 24 April 2023)) for bibliometric analysis and visualization analysis of 442 data points. Chiplot (https://www.chiplot.online (accessed on 24 April 2023)) was used to analyze the annual volume of publications and citations. All data were exported as a “Tab file” and uploaded to VOSviewer for organization cooperative network analysis, journal analysis, author analysis, keyword analysis, and reference co-citation analysis. The online website https://charticulator.com/app/index.html (accessed on 24 April 2023) was used to analyze the number of different countries and cooperation networks.

## 5. The Technology of 3D Printing

**Figure 7:**
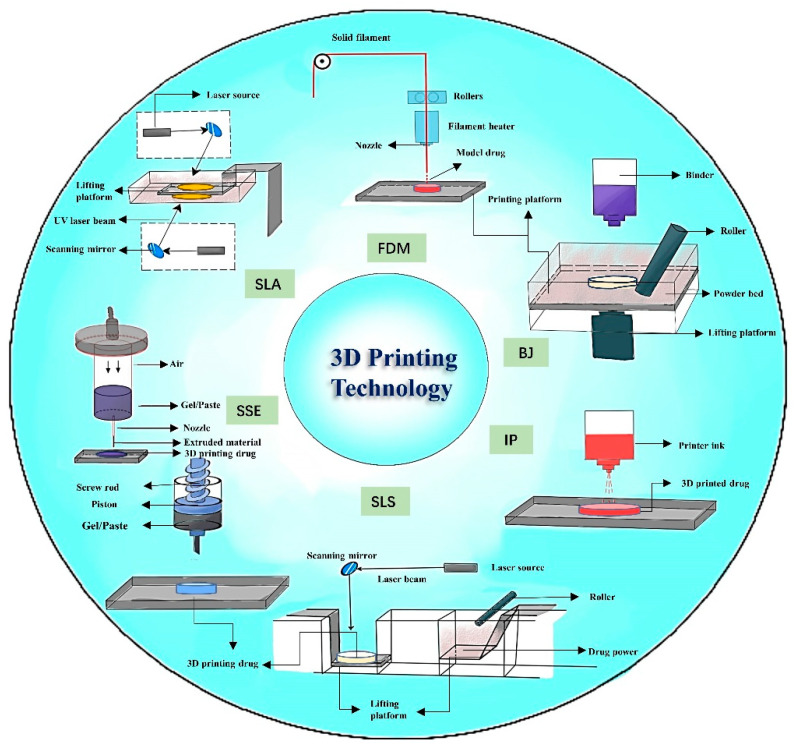
Diagram of different 3D printing technologies.

As a popular new technology in the field of pharmaceutical preparation, 3D printing has attracted a great deal of attention from researchers. In the literature we reviewed, we learned from the above analysis that most 3D-printed personalized drug formulations use FDM technology. The other 3D printing technologies used include stereoscopic lithography (SLA), direct powder extrusion (DPE), inkjet printing (IP), selective laser sintering (SLS), semi-solid extrusion technology (SSE), and binder jet 3D printing (BJ). This section highlights these 3D printing technologies and their advantages and disadvantages (Table 4), and a corresponding technical schematic is shown in Figure 7. Meanwhile, the commonly used 3D printing materials and functions are listed in Table 5.

### 5.1. Fused Deposition Modeling (FDM)

In FDM, a high-temperature nozzle is used to heat a polymer filament containing a drug produced via hot melt extrusion (HME) until reaching the point of semi-liquid extrusion, and the result is then cured on a tectonic plate and formed into a preset geometry [35]. FDM technology also allows for multiple nozzles to be used to print simultaneously and extrude different materials independently. This technology has become the most popular way of 3D printing items used in personalized medicine because of the small equipment, low cost, excellent mechanical strength, high utilization efficiency, and fast printing with which it is associated [36,37]. However, due to the high printing temperature involved in FDM, the applications of some heat-sensitive drugs are limited. In the published literature, most printing temperatures are 135–230 °C [8,38], so the polymer employed needs to have high temperature resistance and good extrusion and printing properties. To date, some researchers have used FDM technology and employed polyvinyl alcohol (PVA) as a polymer to print sustained-release amino acid salicylic acid tablets [7], sustained-release prednisolone tablets [11], etc. There have also been studies in which PVA has been used as a polymer to successfully print drug-carrying filaments of different drug concentrations and make them into tablets [39]. Poly (2-ethyl-tetraoxazoline) [PETOx], which is a new water-soluble polymer, was used as a polymer for the study of 3D-printed controlled release tablets [40]. In one study, hydroxypropyl methylcellulose (HPMC) and polyethylene glycol (PEG) were simultaneously used as polymers for the printing of verapamil pulse tablets, and the results indicated success [41]. In addition, Poly(lactic acid) (PLA) [42], ethyl cellulose (EC) [43], polyvinylpyrrolidone (PVP) [44], Eudragit^®^ [38,45,46,47,48], hydroxypropyl cellulose (HPC) [47,48], etc., have been used in recent years, and many researchers have combined HME with FDM technology to print medicines, providing a flexible platform for personalized medicine products [43,49,50,51,52,53,54].

### 5.2. Stereo Lithography Appearance (SLA)

SLA is a 3D printing method in which lasers and resins are used. It features the use of a single laser aimed at specific points to cure the resin and solidify patterns [55]. This technique requires a large amount of energy from the laser used and is affected by the corresponding power of the light source, the scanning speed, the exposed material, and the polymer and photoinitiation dose [30,36,55]. Presently, the most commonly used photo-initiator is diphenyl (2,4,6-trimethyl-benzoyl) phosphine oxide (TPO) [56]. Unlike FDM technology, SLA technology can be used to print at room temperature and is suitable for printing formulations with heat-sensitive drugs (which can reduce drug degradation) [6,56]. For example, one group of researchers dissolved a drug in different mixtures of polyethylene glycol diacrylate (PEGDA) and polyethylene glycol (PEG), which were then cured in the presence of a laser beam to prepare a drug delivery device (with a nasal shape) with higher resolution and higher drug loading (1.9% *w*/*w*) and no drug degradation compared to a device prepared via FDM [57]. In addition, the authors of another study used SLA technology to print multilayered pills containing six drugs with different drug release curves; this was the first use of SLA printing to develop a multidrug treatment, promoting a new era in personalized medicine [30]. However, due to the limited choice of biocompatible polymers and photo-initiators and the high cost of the system, the development of drugs using this technology has been limited. With the improvement of modern science and technology, the further development of SLA technology in the future will allow it to overcome restrictions and to be more widely used in the 3D printing of items used in personalized medicine.

### 5.3. Inject Printing (IP)

Inkjet 3D printing involves a liquid material being specifically and selectively sprayed onto a substrate and then solidified to produce a specific drug. The ejection process consists of three stages: (1) droplet generation, (2) droplet deposition and substrate interaction, and (3) solidification. This technique is also known as powder drip (DOP) [37]. The solidification mechanism can occur in different ways, including through solvent evaporation UV curing or via a chemical reaction [58]. The advantages of this technology are mainly that it reduces the number of steps required in manufacturing personalized tablets and that it allows for drug release to be controlled freely and flexibly, with high accuracy, good reproducibility, and low cost [36]. FDM and inkjet printing can be combined to print drugs at the same time. In a study by Eleftheriadis, G.K. [19], a heat-unstable model drug, was printed on an FDM substrate via inkjet printing due to the high temperature of FDM, and after evaluation, the method was found to be feasible. The current challenge is ensuring the development of printable inks because the physical and chemical properties of ink can have a great impact on printing [36,58].

### 5.4. Semi-Solid Extrusion (SSE)

SSE is one example of a 3D printing technology. In this process, semi-solid formulations such as gels or pastes are squeezed out of nozzles through pistons and deposited layer by layer on a build board to obtain the desired drug [59]. The main advantages of SSE are its low printing temperature and operational simplicity, causing it to receive great attention in the field of 3D-printing-based personalized medicine [5,13]. Filipa Dores et al. successfully produced theophylline tablets using PVP and PVA as pharmaceutical ink in combination with other excipients at 65–100 °C [60], and Johannesson, J. et al. printed solid lipid tablets containing the poorly water-soluble drug fenofibrate using SSE at room temperature [61]. The printing temperature of SSE is significantly lower than that of FDM. In addition, its drug load is also high. S.A. Khaled et al. successfully printed (80% *w*/*w*) paracetamol oral tablets by applying an extrusion-based 3D printer to a premixed water-based paste formulation [62,63]. However, to achieve the best process, in addition to the advantages, we must also consider issues such as a material’s viscosity. Compared with other technologies, SSE has a lower print resolution, which may affect the development accuracy of the print, but this actually improves the printing speed [13].

### 5.5. Selective Laser Sintering (SLS)

SLS is a powder bed fusion technology in which a powder bed is used to build 3D objects, bond powder particles together using a laser, and draw specific patterns. The polymer materials used in this technology are mainly thermoplastic polymers [23,24]. Compared to other 3D printing technologies, the preparation process for SLS technology is solvent-free, so the process does not require additional drying steps and allows for rapid printing; in addition, easily hydrolyzed drugs are more stable [23]. Due to the high resolution of the laser beam used in the process, SLS can be used to design complex and elaborate dosage forms. However, drugs that are unstable against light and heat are prone to drug degradation in this process. Therefore, light and heat are among the factors limiting the development of this process. This process requires a large amount of powder material, which can increase losses and costs if the material is not handled properly [23,24,64,65].

### 5.6. Binder Jet (BJ)

Binder jet 3D printing is also a 3D printing technology. It is based on a process in which an adhesive is sprayed onto a powder with tiny ink droplets through a nozzle to bond it, forming a 3D structure [66,67]. Similarly, this technology can be used to develop personalized formulations and create accurately targeting and simplified drugs with great accuracy and flexibility and lower drug development costs. However, there are still great challenges in terms of printing equipment and process parameters [68]. The adhesive used is usually composed of organic solvents, and in terms of medication safety, the residue of the solvent must be evaluated [66]. Since BJ 3DP is limited by the thickness of the powder layer, a “coffee ring” effect may occur [69,70], so obtaining high-resolution objects can also be challenging.

### 5.7. Direct Powder Extrusion (DPE)

This technique involves adding prepared mixtures, granules, or abrasives to a printer and extruding them through nozzles [34], allowing a drug to exist in an amorphous state. This method enhances the absorption and dissolution of the drug and has a certain effect on the masking of the drug’s odor. At the same time, the process of the hot melt extrusion of filament yarn is avoided, and the shortcomings of the insufficient mechanical properties of filament yarn are avoided; thus, mixtures that cannot be printed via FDM can be extruded [15,16]. In addition, Magdalena Kuźmińska et al. proposed a method for reducing the duration of the drying step after printing, thereby reducing the risk of drug hydrolysis [71]. This work contributed to the efforts of developing a simplified, facile, and low-cost 3D printing technique for the small-batch manufacturing of bespoke tablets in which the use of high temperatures and post-manufacturing drying steps is circumvented.

**Table 4:** Advantages and disadvantages of different technologies.

Technologies	Advantages	Disadvantages	Print Temperature	References
FDM	Small pieces of equipment, low cost, high mechanical strength, high efficiency, fast printing speeds (15~90 mm/s); generates amorphous solid dispersed filaments as well as amorphous forms of insoluble drugs.	High printing temperatures lead to degradation of thermal drugs; lack of suitable polymer materials.	High temperature (135~230 °C)	[36,37,38]
SLA	Low printing temperature reduces degradation of thermal components; high resolution and high printing accuracy.	UV-initiated polymerization may lead to drug polymerization; limited choice of biocompatible polymers and photo-initiators and high system costs; slow printing speeds.	Room temperature	[30,36,55,56,57]
IP	Simple production steps, high precision, and low cost; can be combined with FDM technology to print drugs.	Printing requires high physical and chemical properties of the drug ink; curing required.	Room temperature	[36,58]
SSE	Room-temperature printing can be carried out without heating, easy to operate, and fast printing speeds.	Low print accuracy; viscosity leads to clogging of easy nozzles; high drug loading capacity.	Room temperature	[5,13,59,60,62]
SLS	No additional drying steps are required; speeds up printing; improves the stability of easily hydrolysable drugs; the high resolution of the laser beam allows for the design of complex and fine dosage forms.	Drugs that are unstable toward light and heat are susceptible to drug degradation during this process; costs may be high.	High energy	[23,24,64,65]
BJ	Accuracy and flexibility are high; low cost.	Composed of organic solvents with safety risks; requires post-processing.	Room temperature	[66,67,68]
DPE	Allows a drug to exist in amorphous state; enhances the absorption and dissolution of the drug; there is no need to prepare a filament.	Limited number of drugs suitable for printing.	Room temperature	[15,16,34]

**Table 5:** Materials used in 3D printing.

Polymers	Characteristics	Functions	Technology Applied	References
Poly (lactic acid) (PLA)	Good biodegradability, biocompatibility, thermoplastic processability, and eco-friendliness.	Filler component; controlled release.	FDM SLS	[72,73]
Polyvinylpyrrolidone (PVP)	Hygroscopic polymer; thermal resistance; biocompatibility.	Filler component; binder; immediate release.	FDM BJ SSE	[74,75,76,77]
Eudragit^®^	Thermoplastic properties; low glass transition temperatures (between 9 °C and >150 °C); high thermostability.	Filler component; various release modifiers; taste-masker agent.	SLS FDM BJ	[20,24,78,79]
Hydroxypropyl Cellulose (HPC)	Good thermoplasticity; solubility is determined by temperature.	Filler component; controlled release; binder.	FDM SLS SSE DPE	[47,48,80,81,82]
Ethylcellulose (EC)	Hydrophobic; thermal characteristics, thermoplasticity, and miscibility with incorporated plasticizers; degradation temperature (Td) is 280 °C.	Release retardant.	FDM	[20,81]
Hydroxypropyl Methylcellulose (HPMC)	Hydrophilic polymer; high melt viscosity; low degradation temperature.	Filler component; various release modifiers; controlled release.	FDM SLS SSE BJ	[68,81,83,84]
Polyvinyl alcohol (PVA)	Water-soluble polymer; melting point of PVA ranges from 180 °C to 220 °C; biocompatibility, non-toxicity, and good mechanical and swelling properties.	Filler component; immediate release.	FDM	[72,81]
Poly (Ethylene Glycol) (PEG)	Water-soluble, biocompatible, and amphiphilic polymer.	Plasticizer; controlled release; PEG derivatives are generally utilized as photopolymerizable (photocurable) polymers.	SLA FDM IP DPE	[21,41,58,82]
Polyethylene glycol diacrylate (PEGDA)	Good biocompatibility; low cost; and water solubility.	Photo-initiator.	SLA	[6,57,85]
2,4,6-Trimethylbenzoyl-diphenylphosphine oxide (TPO)	High biocompatibility and excellent transparency.	Photo-initiator.	SLA	[6,86]
Polycaprolactone (PCL)	Semi-crystalline, biocompatible polyester; melting point of 55–60 °C and Tg of −54 °C; low in vivo degradation; low tensile strength	Filler component.	FDM	[74,77]

## 6. Application of 3D Printing

### 6.1. The Development of Different Dosage Forms

#### 6.1.1. Tablets

**Figure 8:**
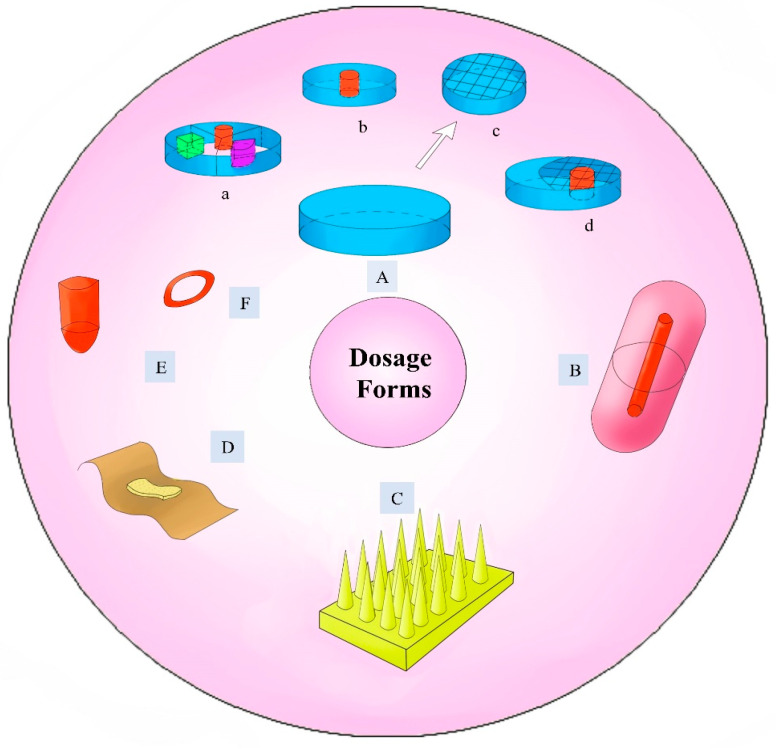
Different drug dosage forms developed via 3D printing. (**A**) Tablet, (**B**) Capsule, (**C**) Microneedles, (**D**) Transdermal patch, (**E**) Suppository, and (**F**) Vaginal ring (a–d are tablets with different internal structures).

Ordinary tablets are accurate with respect to measurement, stable in quality, convenient to consume, and low in cost. As a result, tablets have also become the most common dosage form for 3D-printed drugs, and some of these tablets are shown in Figure 8A. Tablets are the focus of the majority of the literature we reviewed. The first 3D-printed drug approved by the FDA, SPRITAM^®^, is also available in tablet form. There are also a variety of types of 3D-printed tablets. Some patients only need one treatment, and the corresponding drugs can be made into single-component tablets [71]. While some conditions require a combination of multiple drugs, 3D printing can be used to make multicomponent tablets, which can reduce the number of tablets patients have to take [87]. In addition, chewable tablets [88], gastric retention tablets [89,90], controlled-release tablets [91,92,93], immediate-release tablets [9,38], sustained-release tablets [7,94], orally dispersible tablets [70], orally disintegrating tablets [12,22,94], and solid lipid tablets [61] have been investigated. In the future, doctors will be able to choose the right tablet according to their patient’s acceptance and a drug’s properties, enabling more personalized treatment.

#### 6.1.2. Suppository

Suppositories (Figure 8E) are solid preparations made of drugs with a suitable matrix for intracavity administration, mainly through the anus or vagina, and sufficient for softening at body temperature and dissolving in secretions [95]. They are not damaged by gastrointestinal pH or enzymes and are suitable for patients who are unable or unwilling to take oral medications [96]. Chatzitaki, A.T. et al. successfully prepared personalized lipid-based suppositories that can encapsulate different amounts of a lidocaine (LID) free base via the pressure-assisted microsyringe method [97]. Similar to this study, others developed an automated 3D printing process. This technology grants one the ability to tailor drug dosages and shapes to a patient’s needs [98]. Some researchers have used a water-soluble polymer (polyvinyl alcohol) as a suppository shell mold to control drug release [99].

#### 6.1.3. Orodispersible Films

Orodispersible films are pharmaceutical preparations that can quickly release active ingredients in the oral cavity and do not require chewing or water, especially for patients with dysphagia [100,101]. In a previous study, FDM technology was found to be a suitable method for the preparation of aripiprazole-containing orally dispersible films. During the preparation process, aripiprazole exists in an amorphous state, which promotes the dissolution of aripiprazole [102].

In addition, Panraksa, P. et al. successfully prepared oral dispersion films containing phenytoin by using hydroxypropyl methylcellulose (HPMC E15) as a film-forming polymer and glycerol and propylene glycol as plasticizers and achieved excellent performance [103]. Sjoholm, E. et al. used different methods of semisolid extrusion 3D printing to produce transparent, smooth but flexible warfarin-containing orally dispersible films [104]. Furthermore, 3D-printed oral dispersion membranes can also be personalized, but the small drug load involved is a problem [29].

#### 6.1.4. Microneedles

Microneedles (Figure 8C) are transdermal delivery systems that combine a transdermal patch and hypodermic needle technology [105]. Compared to traditional injections, microneedles are safer and painless [106]. The currently used microneedle-based drug delivery system has also attracted worldwide attention [107,108]. However, the precise manufacture of microsized microneedles using conventional methods is difficult. Nevertheless, 3D printing, as an emerging digital technology in the field of personalized medicine, overcomes these difficulties and provides the ideal microneedle for personalized customization [109,110]. In their study, Sirbubalo, M. et al. analyzed the different techniques, parameters, and properties of 3D-printed microneedles [111] to provide a reference for future studies. At present, 3D-printed high-precision microneedles are still limited by materials and printing parameters [112]. Continued efforts of researchers are needed to promote the development of 3D-printed microneedles to contribute to personalized drug preparation.

#### 6.1.5. Implants

Implants are devices that bind to drugs within polymer matrices and are placed inside the body to deliver a drug [113]. Because an implant incorporates a drug and delivers it directly to the site of action, systemic toxicity is reduced, and the therapeutic effect is improved. In addition to the strict requirements for 3D printing methods and parameters, implant materials also have strict biocompatibility requirements [95]. Yang, Y.T. et al. [114] successfully studied ibuprofen-containing polycaprolactone-chitosan delivery implants using HME and FDM technology to control drug release rate by changing the structures and shapes of plants. This study is fully available for personalized drug delivery. In addition, 3D-printed implants have a wide range of applications in tissue regeneration engineering, customizing regenerative drugs according to a patient’s specific characteristics [115,116,117]. 

#### 6.1.6. Other Dosage Forms

In addition to the pharmaceutical dosage forms discussed in this section, other interesting forms have been studied by researchers. Atheer Awad et al. utilized SLS 3DP to prepare 3D-printed multiparticles containing acetaminophen [118]. Some researchers have improved the traditional capsule structure and developed multi-compartment capsule devices (Figure 8B) with different doses or formulas to achieve multiple release effects [119]. Another example is the study conducted by Jingjunjiao Long et al. [120] on the viability of employing 3D printing technology for developing biopolymer hydrogel wound dressings (Figure 8D). There are also 3D-printed vaginal rings (Figure 8F) that are meant to individually treat vaginal disorders. 3D printing can be used to select the right dosage form and prescribe a precise dosage according to a patient’s treatment needs, enabling precise and personalized treatment. In the future, it will be used in an increasing number of dosage forms.

### 6.2. Personalization of Drug Release

Drug release rates can be adjusted via 3D printing by changing the drug formulation and a drug’s geometry, surface-area-to-volume ratio, and fill density to achieve rapid or extended release [48,121,122,123]. By devising diverse printing structures [124,125] and modifying auxiliary materials [38], it is possible to achieve a desired release rate that caters to individual patient needs.

#### 6.2.1. Rapid Release of the Drug

Immediate-release tablets are the most common products administered in oral dosage forms. Since they can disintegrate in the stomach, their active ingredient (API) is released, allowing for rapid treatment. In one study, loratadine was used as a model drug to prepare ten different formulations with crospovidone and croscarmellose as superdisintegrants, mannitol as a pore-forming agent, and polyethylene oxide-N80 and hydroxypropyl cellulose-EF as polymeric carriers. The release rates of all the drug preparations within 30 min were 86.1–96.9% and surpassed the standard of the FDA’s rapid release solid oral dosage form by 80% [126]. Similarly, Allahham et al. successfully printed orally disintegrating printlets containing ondansetron; after in vitro dissolution, they exhibited fast disintegration and released more than 90% of the drug in 5 min. In this study, ondansetron was first incorporated into drug–cyclodextrin complexes and then combined with the filler mannitol [22]. In addition, other researchers have also conducted similar experiments to demonstrate the feasibility of 3D printing fast-release drug formulations.

#### 6.2.2. Extended Release of a Drug

Sustained release formulations can minimize frequent daily dosing and reduce side effects caused by fluctuations in the plasma concentration of a drug, thus improving patient compliance and treatment outcomes. HPC, Eudragit (RL PO), and PEG are traditionally used as slow-release products. In the cited study, novel formulations of 3D-printed extended-release preparations containing all three polymers at the same time were developed and optimized. Different slow-release modes could be achieved by changing the concentrations of HPC and Eudragit [127]. In another overview [52], the author presented some polymerizations and formulations of extended-release preparations prepared using an HME-FDM combination, providing a reference for the development of 3D-printed sustained-release preparations.

In addition to the above two single-release rates, researchers have developed a dual release rate for combi-pills. In a study, the authors used SSE and FDM technology to produce tranexamic acid (TXA) fast-release and indomethacin (IND) slow-release combination pills for the first time [128]. The results showed that the matrix polymer can significantly affect the drug release rate, and the addition of HPMC can change the fast release into a TXA slow release. In another study, atenolol, pravastatin, and ramipril were mixed with an HPMC and extruded into a segmented compartment made of cellulose acetate to form a sustained-release compartment. Aspirin and hydrochlorothiazide were mixed with other excipients such as disintegrating agents and extruded directly on top of the sustained release compartment to obtain a rapid release compartment [129].

In addition to changing the drug release rate by changing the substrate or drug formulation, other studies have shown that the surface area/volume ratio affects the drug release rate [33]. Under the condition of a constant surface area, the larger volume of a geometric shape increases porosity, resulting in faster drug release. The smaller the SA/V, the faster the drug release rate. A study has shown that SA/V can be used to predict the mean dissolution time (MDT) and drug release rate [123]. In the future, based on the optimal drug release rate for a patient, the selection and appropriate SA/V and drug geometry will make treatments more accurate and promote personalized patient treatment.

## 7. Conclusions and Outlook

Three-dimensional printing can precisely control drug dosages, change drug dosage forms and geometries, control drug release speed, improve patient compliance, reduce drug toxicity, etc., and with these advantages, it stands out in the field of personalized medicine. Our research using visual analysis also shows the potential of 3D printing in the field of personalized pharmaceutical preparations. 

In the last decade, 3D printing technology has led to significant breakthroughs in the field of medicine, especially personalized medicine. For instance, the use of water-soluble suppository shells with different structures to achieve different drug release curves, the development of 3D-printed fudge suitable for children’s medicine, and the use of color jet 3D printing technology (CJ-3DP) to produce colorful cartoon pediatric formulations have greatly improved the acceptance of medicine by children. A multilayer structure (polypills) containing six drugs was printed using SLA technology to achieve multidrug therapy and reduce doses. Currently, many companies are working to apply 3D printing technology to drug production. For example, FabRX Ltd., which is more focused on developing personalized pharmaceuticals, has made the world’s first pharmaceutical 3D printer for personalized medicines. 

Although there has been much drug-based research into 3D printing, it has been limited to the laboratory stage and has not been brought to market. Firstly, the lack of printing materials is one of the main bottlenecks. The adaptation between different technologies and printing materials plays an important role in printability and biocompatibility. Therefore, the development of biocompatible, printable, low-cost, and less-toxic printing materials is currently the main task. Secondly, there are no fixed guidelines for the regulation of 3D-printed drug dosage forms, and there is a need to address regulatory issues as soon as possible; it is also important to keep updating 3D printing technology to increase printing speeds and improve resolution and to reduce the cost as much as possible. Therefore, 3D printing is not yet available for large-scale drug development and manufacturing. However, we believe these problems will be solved in the future. Once these issues are resolved, the pharmaceutical industry will usher in a boom period, with an increasing number of pharmacies and hospitals owning 3D printing equipment and utilizing 3D printing technology to enable on-demand printing and personalized treatment, bringing the pharmaceutical industry into a new era of innovation. In addition, in the Web of Science Core Collection, studies on 3D-printed herbal preparations, such as those used in Chinese medicine, Korean medicine, etc., were not found. In other databases, there are only a few mentions. Due to the complex chemical composition of herbal medicine and the different molding mechanisms of various preparations, excipients suitable for 3D-printed herbal medicine preparations need to be developed. In the future, with the advancement of 3D printing technology, the 3D printing of herbal preparations will continue to improve and advance, greatly promoting the personalized customization of herbal medicine preparations.

## Figures and Tables

**Table 1:** The top 10 journals with the most contributions.

Rank	Journals	Papers	IF (2022)	JCR	Publisher
1	*International Journal of Pharmaceutics*	109	5.8	Q1	ELSEVIER
2	*Pharmaceutics*	74	5.4	Q1	MDPI
3	*Journal of Controlled Release*	18	10.8	Q1	ELSEVIER
4	*AAPS Pharmscitech*	14	3.3	Q2	SPRINGER
5	*European Journal of Pharmaceutics and Biopharmaceutics*	14	4.9	Q1	ELSEVIER
6	*Advanced Drug Delivery Reviews*	12	16.1	Q1	ELSEVIER
7	*Current Pharmaceutical Design*	12	3.1	Q3	BENTHAM SCIENCE PUBL LTD
8	*Journal of Drug Delivery Science and Technology*	12	5.0	Q2	ELSEVIER
9	*Journal of Pharmaceutical Sciences*	12	3.8	Q2	ELSEVIER
10	*European Journal of Pharmaceutical Sciences*	11	4.6	Q2	ELSEVIER

**Table 2:** Top 10 institutions with the most publications.

Rank	Institutions	Country	Papers	Citation
1	University College London	The United Kingdom	52	5898
2	FabRx Ltd.	The United Kingdom	47	5673
3	University of Santiago De Compostela	Spanish	25	1867
4	University of Cent Lancashire	The United Kingdom	16	1994
5	University of Nottingham	The United Kingdom	14	1578
6	Aristotle University of Thessaloniki	Greece	11	297
7	University of Texas Austin	The United States	11	479
8	Abo Akademi University	Finland	10	525
9	University of Mississippi	The United States	10	605
10	National University of Singapore	Singapore	9	392

**Table 3:** Top 10 references with the most be cited.

Rank	Cited References	Year	First Author	Citation Frequency
1	Effect of geometry on drug release from 3D-printed tablets	2015	Goyanes, A.	154
2	3D printing of modified-release aminosalicylate (4-ASA and 5-ASA) tablets	2015	Goyanes, A.	146
3	Fused-filament 3D printing (3DP) for fabrication of tablets	2014	Goyanes, A.	142
4	3D printing of five-in-one dose combination polypill with defined immediate and sustained release profiles	2015	Khaled, S.A.	138
5	Fabrication of extended-release patient-tailored prednisolone tablets via fused deposition modelling (FDM) 3D printing	2015	Skowyra, J.	131
6	Emergence of 3D Printed Dosage Forms: Opportunities and Challenges	2016	Alhnan, M.A.	118
7	3D printing of Medicines: Engineering Novel Oral Devices with Unique Design and Drug Release Characteristics	2015	Goyanes, A.	116
8	3D printing of tablets containing multiple drugs with defined release profiles	2015	Khaled, S.A.	116
9	Desktop 3D printing of controlled release pharmaceutical bilayer tablets	2014	Khaled, S.A.	111
10	A flexible-dose dispenser for immediate and extended release 3D-printed tablets	2015	Pietrzak, K.	111

## Data Availability

The data used to support the findings of this study are included within the article or are available from the corresponding author upon request.
